# Health and Nutritional Beliefs and Practices among Rural Elderly Population: An Ethnographic Study in Western Spain

**DOI:** 10.3390/ijerph17165923

**Published:** 2020-08-14

**Authors:** Borja Rivero-Jiménez, David Conde-Caballero, Lorenzo Mariano-Juárez

**Affiliations:** 1 Department of Computer and Telematic Systems Engineering, Polytechnic School, University of Extremadura, 10003 Cáceres, Spain; 2 Department of Nursign, School of Nursing and Occupational Therapy, University of Extremadura, 10003 Cáceres, Spain; dcondecab@unex.es (D.C.-C.); lorenmariano@unex.es (L.M.-J.)

**Keywords:** anthropology, aging, nutrition, ideologies, health, rural areas, Extremadura (Spain)

## Abstract

Background: Demographic transition is causing an increasingly aged society, which has a significant impact on public health strategies. Increases in the size of the elderly cohort create a wider stratification and pose specific challenges. Nutrition and diet are one key issue. This study aims to describe food-related practices, beliefs, and representations of non-institutionalized older adults in rural communities in Extremadura (Western Spain). Method: The ethnographic-based fieldwork was conducted from January to July 2019. Empirical material was collected through different research relationships (semi-structured interviews and informal conversations) and direct observation in various locations in Extremadura―involving a variety of agents associated with different aspects of the nutritional process. Results: Data analysis revealed four major themes: (1) Limitations on choice and quality of food available; (2) food preferences and cooking methods; (3) the role of nostalgia in the construction of taste preferences; and (4) perceptions of what “healthy” food is and how respondents relate to the advice provided by health professionals. Conclusions: Nutritional choices among the group studied presented significant differences from medical advice―which was seen as a series of “bans” that did not carry enough authority to alter the symbolic value attached to their traditions.

## 1. Introduction

As with many other countries, Spain is undergoing a process of demographic transition [1], characterized by an increasingly aged population. The pressure of this new social reality on healthcare management strategies is immense. Projections in European countries estimate that, by 2064, 33% of the population will be over 65 years old [2], while the dependency ratio for that same date will double present levels (30.5% as of 1 January, 2018) [3].

Those values are particularly significant in countries like Spain, where average life expectancy at birth has doubled since the first half of the 20th century [4,5]. As of 1 July, 2019, those 65 years and older make up 19.49% of the whole population [6]. It is estimated that by 2064 this percentage will be over 38.7%, with 222,000 individuals being 100 years and older―in contrast to the present value of 12,000 individuals [5]. In Spain, aging is particularly acute in rural areas, where the processes of rural–urban migration that have taken place since the 1960s have caused an increasing depopulation―particularly among the younger generation [7].

The consequences of these demographic dynamics have been the focus of regular debates, since this is one of the greatest challenges of the 21st century [8]. The impact of these dynamics on issues such as labor relations, pension policies and models, family policies, education or labor force participation is beyond doubt. However, there are also consequences on the social sphere, affecting family structures, care strategies, and the welfare and public health systems [9].

Diet and nutrition among older adults is one of the main challenges in public health. In recent years this has started to be taken into account as a crucial aspect of the development of public health policies, in line with the recommendations of the World Health Organization and the European Union [10]. In Spain, the “Estrategia Nacional de Personas Mayores para un Envejecimiento Activo y para su Buen Trato 2018–2021” (National Strategy for an Active Aging and Fair Treatment of the Elderly 2018–2021) is an example of these new kinds of policies [11]. From this age-stage several health risks can start to appear, ranging from dysfunctions when chewing, swallowing or ingesting food [12,13] to eating habits that can cause excess weight [14]―often as a consequence of the nutritional quality of dietary patterns [15]. Further complications are related to how meals are cooked [16], as well as to factors such as social isolation and lack of a support network [17]―all of which can, particularly among older men, become barriers to a healthy diet [18]. Besides, structural factors can impose additional obstacles to the access to and supply of food [19]. These can range from the resources necessary to physically access the food system, to the cost, quantity and quality of the choice of products available.

Given their relevance from a nutritional viewpoint, these issues have frequently been addressed in the specialized literature, with a focus on malnutrition [20,21,22], obesity [23] or the quality of the dietary patterns among older adults [24,25]. Numerous studies have also established links between the consumption of certain foods and health issues. However, evidence is more scarce when we examine the habits and representations of non-institutionalized older rural adults [26]. It has been suggested [27] that the units used to analyze dietary patterns from a cultural viewpoint are very different from those used by nutrition specialists, although both are crucial [28]. Any planned public health intervention needs to be cognizant of these issues.

A better understanding of the dietary preferences of older persons can help improve their quality of life. Existing evidence has emphasized the importance of knowing why certain foods are either accepted or avoided. This could help improve the dietary recommendations provided [29], in particular in places such as rural communities, which face specific vulnerabilities [30]. Beliefs, representations, and ideology are key dimensions that mediate dietary choices. It is important that medical advice on modifying certain habits in order to improve health indices is mindful of the meaning and significance that particular choices hold for these actors [31,32]. This study aims to describe some of these representations and the symbolic construction of choice in an under-studied group.

## 2. Materials and Methods

### 2.1. Study Design, Setting, and Participants

This is a qualitative ethnographic study, based on the classic premises and tools of ethnographic fieldwork [33,34]. Data were collected from January to July 2019. Various research relationships were established in order to gather empirical material, such as semi-structured interviews, informal conversations, and direct observation in different places―such as the kitchens and pantries of the participants, places where groceries were acquired, and health and leisure centers. The study was based on the analysis of the discourse of the interviews and the observation made in the spaces. This has allowed a better description of how people understand and comprehend phenomena related to the experience of “eating”: “what” they experienced and “how” they experienced it. In this way, we were able to describe and understand the meaning that the participants gave to their reality and their experiences.

**Figure 1:**
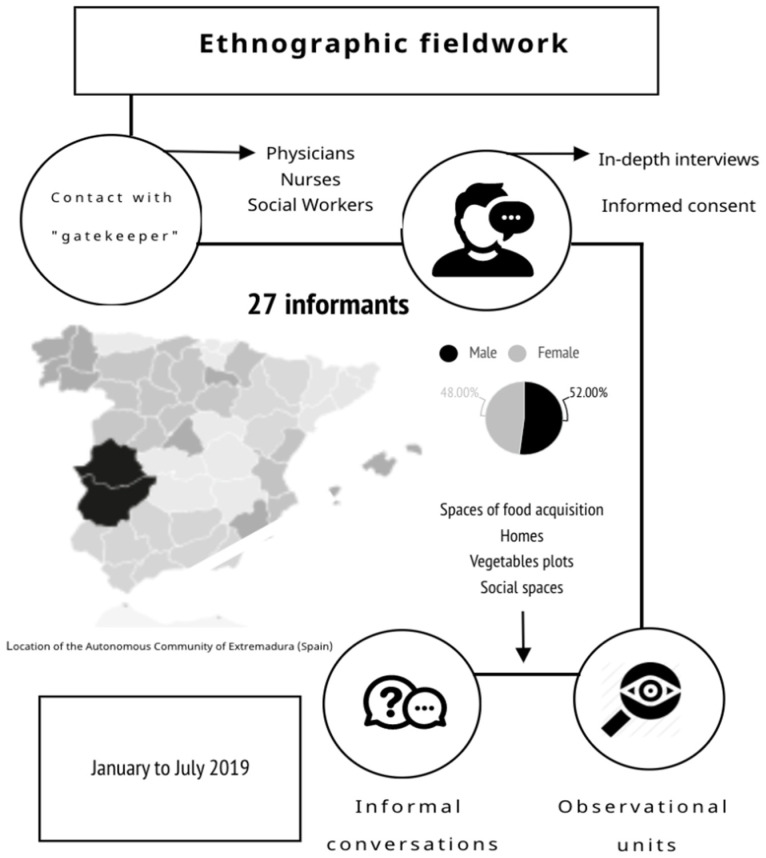
Framework of ethnographic fieldwork.

The participants were residents in seven rural localities in the Autonomous Community of Extremadura (Spain). Extremadura is an autonomous community located in South-Western Spain. Internally divided into two provinces, Cáceres and Badajoz, it borders with Portugal. With a population of only 1,065,575 in 2020 (2.23% of the total Spanish population), of which a significant percentage live in rural communities, its economy is mostly agrarian. Population density is very low―26 inhabitants per square kilometer, while the national average is 92 inhabitants per square kilometer. The percentage of elderly population in this region is above the Spanish average, at 20.64% in 2019, with an aging index of 140.84 and a dependency ratio of 54.56 according to data from the Spanish National Institute of Statistics [6]. The selection of different localities within this region was intended to widen the sample, and it was based on key socio-demographic criteria, i.e., mean age of the population, population density, localities with low population (under 1000) or rurality ratio (Figure 1).

### 2.2. Participant Recruitment Procedure

Participants in different localities were recruited following a purposive, non-probabilistic sampling method. This was preceded by interviews with local medical staff (physicians and nurses) and social workers in each of the localities under consideration. These were informed about the research goals and the participant profile that the study was interested in.

Criteria for inclusion were to be over 65 years old, resident of the localities considered, not institutionalized in any care facility, and with intact cognitive abilities. Those with cognitive impairments and those who did not give their consent to the study or to being recorded were excluded from the study. Medical staff and social workers acted as primary gatekeepers for the study [34], contacting potential participants, explaining the research goals and the structure of interviews, and arranging dates and times for the latter in the event of their acceptance. Once this stage had been cleared, four researchers carried out the interviews―which took place in the participants’ own homes, thus opening them as observational units.

### 2.3. Ethical Considerations

The Bioethics and Biosafety Committee at the University of Extremadura provided the ethical approval for this project (61/2020). All subjects signed a consent form in which they were informed of the objective of their participation in the study and its research goals. They were also informed that they could discontinue their participation at any time without consequences. Confidentiality of the participants’ personal data was guaranteed throughout the whole of the research process. The first author codified the participants’ personal data, and the rest of the research team worked only with those coded identities. The study was conducted in accordance with the ethical principles outlined in the Declaration of Helsinki and the Belmont Report.

### 2.4. Data Sources, Instruments, and Data Collection Processes

The main source of data was semi-structured, in-depth interviews [35,36], which were conducted in the participants’ own homes. This ensured optimum conditions of privacy and comfort for the interviewees. To start with, 76 individuals from the localities chosen for the study were asked to fill in a custom questionnaire. This questionnaire was then used as a guide in the design of the interviews. It included questions about the preparation and consumption of different foods. Finally, a total of 27 respondents were interviewed, and 64 h of recordings were obtained. Of the interviewees, 52% were male and 48% were female. Their mean age was 79.5 years. Of all the interviewees, 65% lived with a partner, 15% lived with an adult son or daughter besides their partner, and 35% lived alone (Table 1).

A research guide was developed, including an introductory section for biographical data and five content sections: (1) Structure of meals, (2) meal preparation, (3) food acquisition, (4) food insecurity, and (5) food and health. The initial interview script was based on the main study goals, in line with our previous research experience on this subject and other studies conducted in similar environments or contexts [37,38,39]. A number of pilot interviews were carried out in order to test the adequacy of the research guide. However, the interviewers were open to explore new subjects not initially considered that could arise during the interviews [40], following an inductive approach. Thus, questions were progressively modified in order to adapt to the new subjects that arose during the respondents’ accounts.

Interviews were backed up with other field research techniques (Table 2), such as informal interviews with different agents in the area―i.e., shop attendants and street sellers, individuals from the area who had not fulfilled the initial inclusion criteria, and key agents; direct observation in the spaces for food acquisition in the chosen localities and in the respondents’ own homes and vegetable plots; and a field diary that provided additional information on specific contexts and issues, allowing us to build a more comprehensive picture of the participants [41,42].

### 2.5. Data Management and Analyses

All interviews were audio-recorded, and field notes were taken while they took place. These were conducted by a team of four researchers with extensive experience in qualitative research and in-depth interviews. The audio recordings were transcribed literally, sometimes with additional information from the field notes. In order to protect the anonymity of the respondents all individual references that could lead to their identification were eliminated. The transcribed interviews were analyzed using the qualitative data analysis software ATLAS.ti (Scientific Software Development GmbH, Berlin, Germany, version 7.5.7 for Windows). Data collected were stored using the Dédalo Intangible Heritage Management platform, an online digital data repository with private access that stores information in a cloud―both original audio- and video-recordings and their transcriptions.

Empirical material was analyzed using the constant comparison method, inductive analysis and triangulation [43,44], also taking into account the idea of “reflexive reflexivity” that is an essential component in the social construction of knowledge [45]. One of the members of the research team individualized the categories identified during the design of the research guide and added those that emerged during interviews, following a deductive–inductive approach [46]. The different categories were presented to the rest of the team, who re-examined the interviews individually first, and then the whole team analyzed and interpreted the interviews again, thus completing the triangulation. The analysis met 31 of the criteria defined in the COREQ (Consolidated Criteria for Reporting Qualitative Research) checklist for reporting qualitative research [47].

## 3. Results

The analysis of the empirical material identified four major themes or categories: (1) Access to food and food supply; (2) food preferences and how food is prepared; (3) the role of nostalgia on dietary patterns; and (4) representations and ideologies about what healthy food is (Table 3). All and each of these categories played a role in the dietary choices of the population group studied.

### 3.1. Food Acquisition

The access to and the supply of food was a recurrent theme in the respondents’ narratives. Respondents often commented on their concern about the difficulties of accessing food―a common issue in rural communities such as the ones in this study, where the provision of certain services can be difficult. These communities are often isolated, and the increasing rural–urban migration has caused the closure of local shops. The youngest often migrate in search of better possibilities. The surviving local shops generally only stock “the simplest of things”, according to one of our respondents―basic supplies useful when they run out of something, but which do not play a major role in the overall process of food acquisition:

“You cannot [shop] on a daily basis [...] because there are no shops left. There were shops before. There was a shop here, a bar. In [name of the locality] there were three or four shops or bars, too. In [name of nearby locality] as well... But there is none left now...” (P2)

It is also important to take into account the physical limitations of the elderly, and the poor communication infrastructure existing in rural areas. Even for those with a private vehicle, additional worries and fears emerge with the idea of driving a car to a supermarket. Finally, the limitations of rural public transport make it an infrequent choice for the elderly to travel to larger populations.

“I used to go very often, but lately I have this leg, I cannot really get to walk. It is bad. Jump in the car, climb down, come back, jump in again. Oh... It is impossible, really.” (P9)

For these reasons, food acquisition usually takes places in farmers’ markets or via supply trucks that visit the localities at certain times and days. However, this poses further limitations on the choice of products that can be acquired. For the eldest in these communities, this is where they acquire most of their groceries. Therefore occasionally, if a product is not in stock, most likely they would have to wait another full week for it. This limitation is particularly clear when it comes to basic products such as bread, but it is even more poignant with the access to fresh fish: none of the shops that we visited stocked any, and only one stocked some frozen varieties. The lack of choice and the gaps in the supply system have a direct impact on the type of food that is consumed. This insecurity is an important source of concern and thus takes a central role in the respondents’ narratives:

“Here, if it wasn’t because they come on Thursdays, the fruit, the frozen products, and the man who comes with the meat, well we wouldn’t be able to eat...” (P4)

Self-provisioning strategies provide a solution for food supply limitations, albeit partial. Most individuals in these communities still have a small patch of land where they grow fresh fruit and vegetables. These can be described as traditional, family-run systems of agricultural production [48], and for centuries they have been an essential source of supply in Spanish rural communities. We conducted direct observations in some of these traditional gardens, which nowadays in addition to their function as a source of fresh produce also provide recreational and occupational values. However, despite many of the respondents owning some land where they could grow produce, these only supplemented a small portion of their diet―fresh fruit, vegetables, and legumes. Although these were seen as “very healthy”, their consumption was limited by the hardships of working on the land at a certain age:

“Because of my effort and determination I still have a little garden down there. Just over a hundred meters from home... And I go there sometimes but look what state I am in, I just dig a trench with a hoe, three meters long, and I have to sit down. So I have a rest. Then I get back up and dig another trench. And so I keep going and I plant potatoes, and frejones [beans], tomatoes, and all of these little vegetables I plant there...” (P19)

It is interesting to appreciate that, in these days where supermarkets seem to paint an image of complete availability, rural elderly populations still consider the food sphere in terms of “what is missing”. Context poses barriers to acquisition and creates inequalities. On the one hand, in purely economic terms, this region has some of the lowest retirement pensions in the country, which limits access to certain products. On the other hand, certain physical health issues such as oral or dental problems can also limit what food can be consumed. Again, this is a recurrent theme in their narratives: “Before we could not eat because we did not have anything, and now that we do, we are not able to.” (P14)

### 3.2. Types of Meals and Their Preparation

When we asked our respondents about the kinds of meals they consumed and how these were prepared, most answers referred to traditional recipes and cooking methods. The *cocido* or *puchero* is a salient example of this. This traditional one-pot stew is prepared with different seasonal vegetables, and it can also take whatever cuts of meat that are available―pork, chicken or beef. In Spain this is an omnipresent dish, an example on how to feed many through the combination of a few humble and economical ingredients―a popular dish that with minimum investment yields a result that is rich, nutritious and very filling [49]. The *cocido* was referred to in several accounts as a meal with multiple qualities; since it contains meat and vegetables it was seen as a “complete meal”, and it was also suggestive of what the respondents considered as “natural”, and thus healthy, food. It is also a very sustainable dish, since it could use any ingredient available, and any leftovers could be re-used again in new recipes.

“I prepare a cocido every fifteen days [...] I put in a small cut of beef, a bone, a piece of chicken to make some stock [...] Then I take the stock out and prepare it. I have it with relleno [dumplings] two or three times, and then sometimes I make croquetas [croquettes], whatever day I feel like it and if there is any of the meat that I had put in or anything else, sometimes the chicken, and I make these natural croquetas.” (P17)

A similar narrative is created around bread. Bread was often mentioned in the interviews, as can be expected from a product that has always been crucial in its meaning, roles and values within the Spanish food culture. For this reason it is seen as a quintessentially symbolic food [50,51], and the “healthiest” one as well―to the extent that even today its absence at the dining table carries, for many, a symbolic invalidation of the act of eating: “For my husband if there is no bread at the table to go with his meal, it is as if he had not eaten...” (P10)

Many of these preferences, however, have recently been subject to changes. Medical recommendations have played an important role in this. Among health professionals, the limitation of the consumption of bread takes a central place: “Bread... well, one bread stick lasts me four days. One of these that cost 80 cents. So... I have to be careful with what I eat, because of my sugar levels...” (P6) This issue is neither novel nor particular to this area―as Contreras [50] has pointed out, bread has gradually given way to other food products. In our study, this happened more frequently among women than among men.

These two items, *cocido* and bread, appeared in almost all of the narratives recorded as central components of the diet. The participants described a basic structure of three main daily meals, in the morning, around midday, and at night. Breakfast, in the morning, consisted of coffee and toasted bread for most respondents. All of them coincided in taking their main meal at midday. For this meal, as mentioned above, some variety of *cocido* was the most frequent option, albeit using different vegetables and cuts of meat. At night the choice was almost always limited to what was considered “frugal” food―eggs, omelets, soup. In many occasions there were references to what were called “knife meals”―bread and cheese or cured meats eaten with bare hands, with the only help of a knife. The need for a frugal supper was usually a medical recommendation and related to health issues, according to our respondents. Some of them also had something to eat mid-morning or mid-afternoon, usually a coffee and pastry (biscuits, a small cake) or a piece of fruit. This, at least, was the case with our female respondents. Male ones, however, admitted as their mid-morning or mid-afternoon snack the traditional *pincho* (a small portion of food served on bread), taken at the local bar with a small glass of wine―although some of them nowadays did this at home instead of going out, due to physical health limitations.

The traditional gender roles were still prevalent, with women almost exclusively in charge of preparing meals, as well as other household chores. For those couples where the woman’s ill health made her unable to take care of cooking, the usual alternative was hiring some external help or receiving assistance from a close relative―a sister or a daughter. However, some men declared being in charge of preparing certain dishes, such as *migas* (a traditional meal of crumbled stale bread fried with other ingredients) or meat stews―dishes traditionally associated with the food eaten outdoors during the agricultural working day.

However, cooking methods seemed to be experiencing a transformation and were more varied now. In contrast to the traditional method, which involved extended cooking times with a pot over a low heat, in most houses it was now common to have meat or fish cooked on a griddle. Again, respondents attribute this change to the health professionals’ insistence that they eat “without oil and with no deep-frying.” (P2) Ovens were almost only used on special occasions, when receiving visitors, or for those foods that were only eaten on holydays. Microwave ovens, which only appeared in rural areas in recent times, were in general only used occasionally―and not at all in some homes, due to limitations in the electrical supply: “The microwave is stronger than the electricity, it trips and [the fuse] blows up.” (P21)

### 3.3. Nostalgia for the Past through Dietary Patterns

For older persons food taste preferences and cravings are closely intertwined with their vital stories, and are often mediated by those flavors that bring back memories of childhood [39,52]. However, sometimes because of barriers to acquisition, and sometimes because of medical recommendations, our respondents’ narratives reflected certain nostalgia for foods that they used to be able to consume, but now could not:

“- Did you use to prepare your stews in a different way?

- Over firewood, over firewood. That was a lot nicer. You would bring your pot with the chickpeas near the fire and you could put anything in it, a leg, or a bit of an ear, because we used to have a *matanza*, and with the *matanza* we had enough [meat] for the whole year...” (P9)

The *matanza* is a pointed example of this. *Matanza* is a seasonal slaughtering of one or several pigs with the aim of making the most of their meat, which often was cured and could last and feed a family for the rest of the year. In Spain the *matanza* used to play a crucial role in the meat supply of families in rural areas―sometimes being almost their only source of meat. These days, however, with the increasing urban migration and the aging of rural population, together with medical recommendations that fatty foods are to be avoided, its value as a self-providing strategy is only residual. Despite this, our respondents’ narratives still included frequent references to the meat available after the *matanza*, which some considered as the “most natural” and the “healthiest”―in clear contradiction to current medical advice.

“These days there is not as much pork belly available as there used to be, we used to do a *matanza* and things like that, but now one does not do *matanza* or anything... “(P3)

“(...) I used to slaughter the pigs here, they weighed over three hundred and sixty kilos and that was enough for the whole year, there was pork belly, there was chorizo sausage, anything, but now it is just the two of us here, what is the point?” (P8)

In addition to the lack of certain foods there was nostalgia for some that were still available, which the respondents were not allowed to eat anymore. Oral–dental problems and the dysphagia affecting some of the older persons made it impossible for them to eat the way they would have liked to. This created a space for nostalgia around certain foods, which was fueled by memories from the days when their health allowed them to eat without limitations.

“Well, before, when I was of such an age that I could go out, I would take some bread to the field, I would go and take some things or others, yes I used to eat bread but since I... Well, I changed my denture, because it did not have... It was bad and I changed it, I hardly eat any bread now because of that. If I eat a small bit... If I do, it is in the summer, with one of these tomatoes we get here from the garden, I eat a bit of tomato, a bit of bread, well... Otherwise no... I do not eat...” (P3)

Besides the nostalgia for certain foods there was also a yearning for the socializing that used to take place around them, which was now lost. In order to cope with this nostalgia, sometimes certain activities are re-signified. We observed, for instance, how supply trucks and farmers’ markets had acquired new relevance as spaces for socialization―a role traditionally associated with the places where food used to be acquired and the communal baker’s ovens in rural areas. On delivery days, there is a time and place set for the “opening”. The eldest start gathering there as if they were attending a meeting. In those communities where the population is diminished and often isolated from each other, these spaces are not just about food―they offer an opportunity for maintaining the traditional bonds with the neighbors.

Another social dimension of food is associated with the act of eating itself. However, the television was now a component of meal times. In particular for those living alone, it made up for the lack of company at the dinner table: “I like the television a lot because I am alone, I have nobody so I switch on my television and it is my companion.” (P21) Living in isolation can sometimes make the act of sitting down for a meal seem pointless. Eating is almost perceived as a chore, something that has to be done but has lost its enjoyable aspects―its preparation and consumption turning into purely mechanical actions. The past, those days when the food and the home were shared with more members of the family, is idealized as a time when food was better despite shortages or even deprivation, and the companionship made the act of eating more meaningful.

“I used to eat better than I do now. I do not know why this is so. You see, there used to be a lot of people at home, so I had to prepare food whether I wanted to or not. And I had to eat. But now, most of the time I say to myself: What am I going to prepare? So instead of preparing dinner I sit down and drink a glass of milk and I leave it at that. So... I have been alone for a long time now...” (P18)

### 3.4. Representations and Ideologies about What Healthy Food Is

Food acquisition, alternative ways of cooking, and the feelings associated with food and eating are all crucial elements that help define dietary patterns among older adults in the communities analyzed. Besides these factors, though, we were also interested in exploring the role of food-related ideologies and the respondents’ beliefs about what is considered healthy food―since dietary preferences can also be an expression of certain ideas and beliefs [53]. Our respondents repeatedly mentioned food “from the olden days”―traditional food―as being more “healthy” and “natural”.

“We do not eat healthy food anymore. For us [healthy eating] is to pick our own potatoes, which are healthy, our own green beans, our zucchini, tomatoes, little lettuces... That was healthier...” (P16)

The food obtained from their own gardens and through the seasonal *matanza* was generally considered as having special qualities and was idealized and perceived as healthier. Thus their opinion was that their diet was not as healthy anymore because, although they still grew some produce and consumed *matanza* meat, this way of producing food was associated with times past. Food produced nowadays, in industrial contexts, generated mistrust as to its health properties, based on suspicions about complex production processes where the food chain was no longer clear, and included elements such as “composite ingredients”, “animal feed” and “sulfates” of unknown origin. It is almost as if the further away from the consumers the food was produced, the less qualities and good properties it maintained. Besides, the current production process was seen as too “rushed”, and it did not allow food to develop the good properties that it had “before”:

“[Meals] were a lot healthier before. They were healthier before because you did everything yourself, you slaughtered the pigs, nothing contained additives, not even the bread... Before, we used to eat and it was mostly vegetables. Perhaps if you had it you would put a bit of chorizo sausage in, or a bit of the pig’s trotter, or a bit of meat and then you would... You pan-fried it with a bit of oil, and paprika and a bit of garlic and that was very nice, with no... But right now it is not, even the beans, they put ammonia or fertilizer in the soil now... It cannot be...” (P26)

“Animals would eat natural things, and then we would put their manure back in the soil, it was completely clean. Now, with animals, you have to buy animal feed. What is in the animal feed? That is how it starts. The products that we eat...” (P19)

The food from “the olden days” was not only perceived as “healthier”, but also as “heartier”, a quality that was missed as well. This is a direct reference to those foods that were consumed during the long hours of hard, physical agricultural work, which required a high calorific content to provide sustenance. The foods that provided enough energy for this work were thus considered “healthy”. The respondents’ narratives were interspersed with comparisons between how food used to be and how it was at the present. As opposed to the “hearty” food of the olden days, which allowed you to do your work, present-day food was considered “weak”, much of it “light” or “low fat”. Besides, whereas in the past food was “healthy” because the consumers controlled the whole productive cycle, nowadays everything was manufactured, which generated mistrust and rejection.

“(...) what one used to eat here was a small portion of bean stew or something like that, with a chunk of bread and a bit of pork belly and with a sip of wine you were ready to go back to work...” (P3)

“That was oil. Unlike the oil now, that you buy a carafe of oil that says “olive oil” but God knows where it must come from...” (P2)

“(...) because what I was told to eat, what with the “cooked ham”, and this, and that... To me, this “cooked ham” that you buy already sliced... That does not give you strength, you cannot eat that if you are working all day, eating just a bit of that...” (P22)

The different representations between the health professionals and the older adults caused ideological discrepancies. The changes and conflicting advice received on the recommended allowances of certain foods, such as pork meat or bread, generated mistrust toward the experts. Our respondents could not understand why the foods that they considered “hearty” and “healthy” were not seen as such by the medical authorities. This effect was increased due to an easier access, through different media, to information about what should or should not be eaten. Some respondents suggested in their narratives that health professionals did not really know what the best choices for healthy eating were. Thus an ambivalent space was created where patients tried to follow medical advice―since it came from educated persons, regarded as worthy of respect.

“I do as the doctor says, because they studied and have a degree. But before they used to say that pork belly and chorizo sausage and all that caused cholesterol but nowadays, they say that pork meat can help regulate cholesterol. So now, I cannot understand what is what...” (P7)

However, the validity of the doctors’ advice was nevertheless questioned and, as a result, sometimes not all of the dietary recommendations were followed in full. That this could have health consequences was generally taken into account, but sometimes it was ignored, depending on personal food preferences. “Some things have to be done but others, no...” (P22) A pointed example of this is the consumption of certain cured meats, as well as sugar and salt―products that are banned in many health conditions, but that still were part of our respondents’ diets.

“I have diabetes as well, and I eat many things that I should not, we are not that old, although of course, I am 65 and he is going to be 67. I measure my sugar levels here at home but [the nurse] now gives us appointments...” (P10)

## 4. Discussion

Our analysis showed the existence of barriers to the access and acquisition of food among older individuals living in the rural areas studied, and how these affected their dietary patterns. Throughout their narratives, many of our respondents coincided in emphasizing their difficulties to access certain foods and how occasionally, if it was possible, they needed to travel to larger towns to do their shopping. Barriers to access are coincidental with limited economic circumstances in certain cases―with retirement pensions in this region being lower than the Spanish average. Self-provisioning is a partial solution to these problems, but often it is not enough. Previous studies have noted similar issues in rural areas. Schoenberg’s [54] research was conducted in a different context to ours, although rural as well. Her conclusions stressed how for certain older adults access to an optimal dietary intake was not possible, which put them at high nutritional risk. Munoz-Plaza et al. [55] noted how some of the older adults in their study had to travel considerable distances or visit multiple shops in order to access their preferred foods, while those with reduced mobility had to accept products of lower quality or manage without them. This suggests that access is a crucial factor in shaping dietary patterns. The results of Whan and Bowden [56] and Smith and Miller [19] were similar to ours and to the studies mentioned above. They recognized the existence of physical barriers to food access and acquisition, and how these posed limitations to healthy dietary patterns among older persons. For instance, the continuous lack of access to fresh produce such as fish among our respondents can lead to substitutional dietary strategies and the consumption of less recommendable alternatives, as happens elsewhere.

Dietary patterns among older adults are thus shaped by availability, but also by personal choice. Delaney and McCarthy suggested that food preferences were influenced by early dietary experiences [52]. Our culinary universes are not just made out of a mass of indistinct macronutrients and calories. Rather, they express a wealth of individual values and conceptions, with preferences and aversions, which contribute since our childhood to shape a dietary identity that is closely intertwined with our cultural memory [57]. Indeed, Rainey et al. [39] pointed out that dietary preferences in older persons were not only based upon childhood familiarity, but could also be influenced by attitudes and beliefs that predated them. Thus the concept of food as it was “in the olden days” acquires a particularly poignant dimension in the construction of culinary ideologies among the elderly. As a result, they always express a preference for the food that they see as “traditional”. In addition, these foods are often identified as “healthy” and “hearty”, since their production process was controlled from beginning to end and provided enough energy to sustain them through their hard working days. By contrast, modern foods have the opposite consideration in almost all counts. Mckie et al. [58] mentioned how the participants in their study considered that the methods of modern food industry resulted in “adulterated” food, thus sustaining beliefs and interpretations that rejected modern food. This critique of modern food production practices as making food lose its “natural” qualities was mentioned in some of our interviews. In line with this, Dye and Carson [59] reported the concerns among older persons about the use of fertilizers in fruits and vegetables. These ideologies and representations, and the nostalgia for the food of the past, play a crucial role in what our respondents chose to eat from what is available. As Vargas et al. [60] indicated, older adults preferred to include in their diet foods that were seen as “fresh” and “natural”, while Cantarero suggested that these kind of beliefs were the main determinants of food preferences and aversions [61]. We believe that the central role attributed to the *matanza* and its by-products by many of our respondents, despite the fact that most of them have not participated in one for many years, might be attributed to culinary ideologies firmly rooted in the past―when the *matanza* was a central pillar of the household diet and one of the main sources of protein and calories for the whole family.

Food self-provisioning in rural communities emerges as a strategy to offset the nostalgia for the “traditional”, “natural”, and “healthy” food that is not generally available, and also to help increase the supply and thus reduce food insecurity. Nowadays, however, self-provisioning has a lesser impact on the diet, primarily because advanced age makes physical work difficult, thus limiting the production. As a result, these activities play a different role these days, adding cultural, social and physical values to their importance for agrarian and ecological processes [62]. Consequently, although existing literature emphasizes the benefits of growing your own produce for the mental and physical wellbeing of the elderly [63,64,65], the results of our study showed a limited impact of these practices on dietary patterns.

The “gender” variable is a crucial issue. Based on the interviews and, above all, the observations, some aspects deserve to be indicated, especially for subsequent analyses that include consumption patterns. In the case of households where the husband and wife live together, the food, almost always prepared by the woman, tends to be the same for both. This could point to the idea that the scientific literature on the subject has pointed out about the skills and knowledge to pre-prepare food acquired during childhood based on gender roles [66,67]. In single-person households, there are also small denotations. Men tend to cook “fried” in the pan and consume sausages, while women can still prepare more traditional dishes in the pot, with less consumption. Similar results have also appeared in qualitative studies on aging, food and loneliness in Spain with respect to food consumption by men [68], or in studies on the greater healthiness of food consumed by women [37]. However, ideas about the benefits of alcohol consumption are clearly segmented. Men tend to consume significant amounts, which are justified by particular interpretations of health professionals’ messages—“doctors say it, one drink can’t hurt”, “that’s good for the heart”. This type of consumption is linked to traditional notions of male identity, and non-consumption as a female trait. This finding is important because, as Boer et al. point out [17], the prevalence of alcohol abuse and dependence is very high among the older population, especially in men, and alcoholism is known to influence nutritional intake. Although not discussed in this text, we have also observed distinctions in the physical activity of each group, which was greater in men.

In the communities studied, diets were shaped by a compromise between availability and preference. The importance of the later confirms the initial hypothesis that social, economic and cultural factors are crucial in the definition of dietary patterns. At the same time, our research emphasizes that medical advice also plays a relevant role in the definition of the dietary patterns of the elderly. The guidelines provided, however, can conflict with their personal preferences, and thus they are not always met. Although some recommendations were accepted, there remained spaces where preferences prevailed over medical advice. Health professionals sometimes insist on the introduction of new foods or alternative cooking methods, which clash with beliefs that can be deeply rooted. It is important to be cognizant of the fact that certain food preferences are established during childhood and carry far-reaching emotional values that are very difficult to change [69]. This is one of the reasons that might explain the difference between what our respondents consider “healthy” and the advice provided by health professionals. Their narratives indicate the existence of a more “flexible” interpretive space for these recommendations, which are marred by certain mistrust toward the veracity of the message. The importance of medical advice, nevertheless, is still appreciated, and thus respondents attempt to abide by it, despite their doubts. This is in line with the findings of Gustaffson et al. [37], who noted that the older adults in their study accepted the importance of the recommendations expressed by health professionals, even though at times they were not comfortable following their advice. That said, while we admit the importance of medical advice in the definition of dietary patterns, it is also relevant to point out something also remarked upon by Smith et al. [70]. They suggested that the dietary patterns that their respondents preferred and with which they identified often conflicted with medical recommendations. Consequently, some respondents admitted that they sometimes ignored or tweaked this advice, despite being aware of the health risks that their actions entailed. Medical advice, then, is sometimes relegated to a secondary position when faced with the pleasure of eating something that is seen as “proper”, without limitations, “traditional”, and thus “healthy and good”.

Certain limitations to our study should be taken into account. First, it might not be easy to make a straightforward extrapolation of our results beyond the population analyzed. Second, there were difficulties in establishing cause–effect relationships from the analysis of the interviewees’ accounts. Third, the limited reproducibility of the study needs to be considered. Despite the scientific rigor and quality of the method used, the application of this study in different contexts and population groups would require the consideration of different scenarios. Finally, the sample used does not reflect the demographics of the senior population in the area studied. The male–female ratio of participants in our study was more balanced than the male–female ratio in the 65 years and older Spanish population, in which females outnumber males.

## 5. Conclusions

Access to food is one of the main barriers to a healthy diet for older adults living in rural areas. The food locally available and self-provisioning strategies are both inadequate and insufficient. In a context of increasingly aged rural communities, eating practices are being compromised by structural factors. This underlines the importance of developing appropriate public policies to improve the food supply in these areas.

Dietary patterns among older adults are defined around availability, but also by their personal choices. Food ideology and nostalgia influence their preferences for “traditional” foods and cooking methods, often conflicting with medical advice. The work points towards ideological differences delimited by gender, which tend to be situated differently with risky nutritional practices that must be taken into account. Dietary patterns are a nutritional issue, but they also have a meaning that is socially, culturally and economically constructed. Future public health policies must be mindful of these dimensions in their design and implementation, adapting medical recommendations to local preferences in each context. Otherwise, medical advice risks conflicting with the weight of tradition and the emotional values attached to certain foods and habits, thus creating a nutritional risk and increasing health problems among the elderly.

## Figures and Tables

**Table 1:** Participants’ profile.

Participant	Age	Gender	Cohabitation Status
P1	78	Female	Living alone
P2	78	Female	Living with a partner
P3	81	Male	Living with a partner
P4	79	Male	Living with a partner
P5	71	Male	Living with a partner
P6	82	Male	Living with a partner
P7	90	Male	Living alone
P8	87	Male	Living with a partner
P9	86	Female	Living with a partner
P10	75	Female	Living with a partner and adult offspring
P11	77	Male	Living with a partner and adult offspring
P12	79	Female	Living alone
P13	65	Female	Living with a partner
P14	67	Male	Living with a partner
P15	85	Female	Living alone
P16	87	Male	Living with a partner
P17	85	Female	Living with a partner
P18	88	Female	Living alone
P19	73	Male	Living with a partner and adult offspring
P20	70	Female	Living with a partner and adult offspring
P21	82	Female	Living alone
P22	83	Male	Living alone
P23	78	Male	Living alone
P24	70	Female	Living with a partner
P25	74	Male	Living with a partner
P26	87	Female	Living with a partner
P27	90	Male	Living with a partner

**Table 2:** Research techniques used in the study.

Research Technique	Empirical Material Obtained
Informal Conversations	Not always recorded, they allowed improvements to the structure of the interviews and provided additional empirical material.
Field Diary	Notes taken during fieldwork, based upon observations and informal conversations. Contextual information and on other issues.
In-Depth Interviews	Based on the model of semi-structured interviews, but designed to include certain content categories.
Observational Units	Spaces for food acquisition; practices associated with food acquisition; places for food storage, preparation and consumption.

**Table 3:** Categories of analysis.

Access/Supply	Food and How It Is Prepared	Nostalgia	The Concept of Healthy
Difficulties of accessing food in rural settings	Structure of meals	Traditional ways of preparing and consuming food	Descriptions in meaningful terms (conscious or unconscious) for the interviewees
Age-related physical and socio-economic limitations	The central role of the *cocido*/*puchero* (traditional stews) and bread	The *matanza* (seasonal pig-slaughtering tradition)	Perception that “everything that is good” is forbidden
Solutions: Farmers’ markets and self-provisioning strategies (vegetable plots)	Meal preparation, gender distinction	Health conditions limit the consumption of certain foods	Solutions: Farmers’ markets and self-provisioning strategies (vegetable plots)
	Traditional and new ways of preparing food	Food acquisition and consumption as socializing spheres	Some changes are accepted (salt), other are re-interpreted
